# Gunshot Wounds of the Abdomen

**Published:** 1899-08-12

**Authors:** 


### GUNSHOT WOUNDS OF THE ABDOMEN.

The discussion on the diagnosis and treatment of gunshot wounds of the abdomen, which occupied the surgical section of the British Medical Association, was interesting chiefly as demonstrating once more the limitations under which military surgeons must of necessity work when dealing with serious surgical cases on the field. Colonel Stevenson said that when a bullet traversed the abdomen it might be assumed that either extravasation of intestinal contents or hæmorrhage, or both, had occurred. A fatal issue was certain in either. The indication was quite distinct, the abdomen must be opened, the intestines sutured, and the peritoneum cleansed; with all of which, so far as civil practice is concerned, we may agree. But when he came to the statistics his contention was hardly borne out. In the Spanish-American war all the cases operated on died. In the Græco-Turkish war one case of hæmorrhage and one of injury to the liver recovered, and one of wound of hollow viscera died; while in the Tirah campaign five cases were operated on and all died. These results were, perhaps, explained by the conditions of military surgery at the front as described by Major Beevor, who spoke of his own experiences in the Tirah campaign, where, he said, all water within access was contaminated by the urine and fæces of men and animals. To cases occurring under such circumstances the experiences of civil hospitals as to abdominal surgery are hardly applicable.

